# The 9th International RASopathies Symposium

**DOI:** 10.1002/ajmg.a.70121

**Published:** 2026-03-16

**Authors:** Pau Castel, Lisa Schoyer, Beth Stronach, Raya Bogdanova, Anton M. Bennett, Jaishri Blakeley, Miriam Bornhorst, Tammy Bowers, Saskia M. Brachmann, Emma Burkitt-Wright, Kathryn Chatfield, Alessandro De Luca, Khalil El-Chammas, Abdul Elkadri, John E. Fortunato, Bruce D. Gelb, Anne Goriely, Karen Gripp, Kassidy Grover, Lindsay Homan, Kenneth A. Kern, Maija Kiuru, Charles (Chuck) Lawson, Yong-Seok Lee, Frank McCormick, Gina Ney, Cristina Nuevo-Tapioles, Sara Pardej, Elizabeth I. Pierpont, Julia Plank, Nancy Ratner, Katherine A. Rauen, J. Elliott Robinson, Les Rogers, Sarah E. Sheppard, Keir Shiels, David Stevenson, Dagmar Tiemens, Matthew Traylor, K. Nicole Weaver, Marielle Yohe, Tamar Green

**Affiliations:** 1 Department of Biochemistry & Molecular Pharmacology, NYU Grossman School of Medicine, New York, New York, USA; 2 RASopathies Network, Pasadena, California, USA; 3 RASopathies Network, Pittsburgh, Pennsylvania, USA; 4 Division of Interdisciplinary Brain Sciences, Department of Psychiatry and Behavioral Sciences, Stanford University School of Medicine, Stanford, California, USA; 5 Department of Pharmacology and Department of Comparative Medicine, Yale University School of Medicine, New Haven, Connecticut, USA; 6 Department of Neurology, Oncology and Neurosurgery, Johns Hopkins School of Medicine, Johns Hopkins University, Baltimore, Maryland, USA; 7 Northwestern University Feinberg School of Medicine, Ann & Robert H. Lurie Children's Hospital of Chicago, Chicago, Illinois, USA; 8 Noonan Syndrome Foundation, Lake Stevens, Washington, USA; 9 Novartis BioMedical Research, Basel, Switzerland; 10 Manchester Centre for Genomic Medicine, Manchester University NHS Foundation Trust, and Division of Evolution, Infection and Genomics, Faculty of Biology, Medicine and Health, University of Manchester, Manchester, UK; 11 Department of Pediatrics and Division of Cardiology, Department of Medicine, University of Colorado Anschutz Medical Campus, Aurora, Colorado, USA; 12 Medical Genetics Laboratory, Fondazione IRCCS Casa Sollievo Della Sofferenza, San Giovanni Rotondo, Italy; 13 Department of Pediatrics, Cincinnati Children's Hospital Medical Center, University of Cincinnati College of Medicine, Cincinnati, Ohio, USA; 14 Division of Pediatric Gastroenterology, Department of Pediatric Gastroenterology, Medical College of Wisconsin, Milwaukee, Wisconsin, USA; 15 Department of Pediatrics, Ann & Robert H. Lurie Children's Hospital of Chicago, Northwestern university Feinberg School of Medicine, Chicago, Illinois, USA; 16 Mindich Child Health and Development Institute and the Departments of Pediatrics and Genetics and Genomic Sciences, Icahn School of Medicine at Mount Sinai, New York, New York, USA; 17 MRC Weatherall Institute of Molecular Medicine, Radcliffe Department of Medicine, University of Oxford, NIHR Oxford Biomedical Research Centre, Oxford, UK; 18 Division of Medical Genetics, Nemours Children's Hospital, Wilmington, Delaware, USA; 19 Division of Experimental Hematology and Cancer Biology, Department of Pediatrics, Cincinnati Children's Hospital Medical Center, Cincinnati, Ohio, USA; 20 Noonan Syndrome Foundation, Sydney, Ohio, USA; 21 Skaggs School of Pharmacy and Pharmaceutical Sciences, University of California San Diego, La Jolla, California, USA; 22 Department of Dermatology and Department of Pathology and Laboratory Medicine, University of California Davis, Sacramento, California, USA; 23 Parent Advocate, Denver, Colorado, USA; 24 Department of Physiology, Biomedical Sciences Seoul National University College of Medicine, Seoul National University, Seoul, South Korea; 25 Helen Diller Family Comprehensive Cancer Center, University of California San Francisco, San Francisco, California, USA; 26 Clinical Genetics Branch, Division of Cancer Epidemiology and Genetics, National Cancer Institute, NIH, Rockville, Maryland, USA; 27 Perlmutter Cancer Center, NYU Grossman School of Medicine, New York, New York, USA; 28 Division of Interdisciplinary Brain Sciences, Department of Psychiatry and Behavioral Sciences, Stanford University School of Medicine, California, US; 29 Department of Pediatrics, University of Minnesota Medical School, Minneapolis, Minnesota, USA; 30 Division of Experimental Hematology and Cancer Biology, Department of Pediatrics, Cincinnati Children's Hospital Medical Center, University of Cincinnati College of Medicine, Cincinnati, Ohio, USA; 31 Division of Genomic Medicine, Department of Pediatrics, University of California Davis, Sacramento, California, USA; 32 Division of Experimental Hematology and Cancer Biology, Department of Pediatrics, Cincinnati Children's Hospital Medical Center; Department of Pediatrics, University of Cincinnati College of Medicine, Cincinnati, Ohio, USA; 33 CFC International, Roseburg, Oregon, USA; 34 Unit on Vascular Malformations, Division of Intramural Research, Eunice Kennedy Shriver National Institute of Child Health and Human Development, Bethesda, Maryland, USA; 35 General Paediatrics, Great Ormond Street Hospital, London, UK; 36 Division of Medical Genetics, Department of Pediatrics, Stanford University Center of Academic Medicine, Stanford, California, USA; 37 Department of Pediatrics, Radboud Institute for Health Sciences, Radboud University Medical Center, Amalia Children's Hospital, Nijmegen, the Netherlands; 38 Think Bioscience, Boulder, Colorado, USA; 39 The Heart Institute and Division of Human Genetics, Cincinnati Children's Hospital Medical Center; Department of Pediatrics, University of Cincinnati College of Medicine, Cincinnati, Ohio, USA; 40 Laboratory of Cell and Developmental Signaling, Center for Cancer Research, National Cancer Institute, National Institutes of Health, Frederick, Maryland, USA

## Abstract

The RASopathies are a group of congenital disorders with overlapping clinical manifestations that are caused by pathogenic germline or early somatic variants that result in the hyperactivation of the RAS/mitogen-activated protein kinase (MAPK) signaling pathway. Given the heterogeneous clinical presentations of these disorders that involve abnormalities across multiple organ systems, multidisciplinary clinical management and progress in scientific research are essential for optimal patient diagnosis and care. The 9th International RASopathies Symposium, a biennial meeting, was organized by the patient advocacy group RASopathies Network and showcased recent discoveries, case studies, and advances in preclinical research. Participants, who included scientists, clinicians, industry representatives, patients, and family advocates, explored knowledge gaps, innovative clinical approaches, and lived experiences of individuals with a RASopathy. Sessions centered around organ systems were introduced with a patient perspective to highlight the burden of disease, continued with presentations from established and early-career investigators. Overall, the RASopathies Symposia serve as a catalyst for sustained community collaboration focused on enhancing patient health and accelerating the translation of discoveries into effective treatments.

## 1 ∣ Introduction

The RAS/MAPK pathway is a highly conserved intracellular signaling cascade that transduces extracellular cues, such as growth factors, into precise cellular responses controlling proliferation, differentiation, survival, and metabolism, among others. Activation typically begins at receptor tyrosine kinases, which lead to the rapid activation of RAS GTPases. Active RAS then engages RAF kinases, initiating a sequential phosphorylation cascade involving MEK and ERK that culminates in the regulation of transcription factors and cytoplasmic targets that reshape gene expression programs and cellular phenotypes, respectively. Tight regulation of this pathway is essential for normal development and tissue homeostasis and, therefore, negative mechanisms that lower signaling output and restore basal activity are crucial (Hibshman and Der 2024; Dutkiewicz et al. 2024).

Germline or mosaic pathogenic variants in components or regulators of the RAS/MAPK pathway cause a group of neurodevelopmental syndromes named the RASopathies. These disorders share overlapping clinical features that reflect RAS/MAPK pathway dysregulation during development, including distinctive craniofacial features, cardiac, lymphatic and other developmental malformations, growth abnormalities, predisposition to malignancies, and cognitive impairment (Rauen 2013; Hebron et al. 2022; Zenker 2022; Tartaglia et al. 2022).

The 9th International RASopathies Symposium was held in Orlando, Florida on July 25–27, 2025, and included 157 inperson and virtual registrants from a broad audience. The agenda brought together basic and clinical researchers, health professionals, individuals with a RASopathy, and caregivers in sessions that addressed particularly challenging or understudied organ systems (e.g., GI, brain, lymphatics), as well as genetic complexities, cancer risk, and drug repurposing. In this review, we share the proceedings of the meeting, highlighting areas of new investigation and concepts to attract early career investigators, principal investigators, and other stakeholders in this fascinating field.

## 2 ∣ Conference Opening Night

The symposium opened with a welcome from conference chairs Pau Castel, PhD (NYU School of Medicine) and Tamar Green, MD (Stanford University School of Medicine) and the meeting organizers Beth Stronach, PhD, and Lisa Schoyer, MFA (RASopathies Network). The session included awards for early career scientists and was followed by a poster session that show-cased 19 scientific posters and nine posters by families living with a RASopathy, namely Noonan syndrome with multiple lentigines (NSML) with a *PTPN11* variant, Noonan syndrome (NS) with a *KRAS* variant, NS with a *PTPN11* variant, Costello syndrome (CS) with *HRAS* variant, cardiofaciocutaneous syndrome (CFCS) with *MAP2K1* variant, neurofibromatosis type 1 (NF1) with an *NF1* variant, and a patient with congenital melanocytic nevus (CMN) syndrome with an *NRAS* variant. The poster session provided a unique opportunity for sharing multi-stakeholder and multidisciplinary perspectives that enhanced understanding and reinforced the urgency to seek improvements in care and treatment.

Following the poster session, Jaishri Blakeley, MD, Johns Hopkins School of Medicine, structured the opening keynote on practical lessons to optimally design and conduct clinical trials with lessons learned from NF1. Dr. Blakeley compared the diverse manifestations of NF1 to other RASopathies, then focused on peripheral nerve sheath tumors, using plexiform neurofibromas (pNF) to represent a compelling therapeutic target. She emphasized that optimal clinical trials require deliberate identification of treatable targets, exploring past successes and failures, built on preclinical models, natural history studies, endpoint development, and disease biology. Drawing from her clinical trial experience with pNF and atypical neurofibromas, Dr. Blakeley illustrated how rigorous trial design can determine success or failure. Using the selumetinib (a MEK1/2 inhibitor) SPRINT trial as an exemplar, she demonstrated how predictive preclinical data, realistic enrollment goals, and endpoints grounded in the NIH NF1 natural history cohort enabled detection of durable tumor shrinkage with acceptable toxicity, supporting the first FDA-approved systemic therapy for pediatric NF1-associated pNF. This success rested on longitudinal natural history data linking pNF growth to pain and functional limitations, volumetric MRI as primary endpoint, and patient-, performance-, and clinician-reported outcomes. In contrast, Dr. Blakeley used the abemaciclib (a CDK4/6 inhibitor) trial for atypical neurofibromas to illustrate how studies falter when enrollment is incomplete, endpoints lack specificity, or missing data undermine analysis. Dr. Blakeley concluded that thoughtful preparation and integrated collaboration and funding, such as that provided by the Neurofibromatosis Therapeutic Acceleration Program, are essential to advance NF1 research translation from preclinical discovery to therapeutic intervention.

## 3 ∣ Session 1: Systems—Feeding and Gastrointestinal Function

This session, moderated by Katherine A. Rauen, MD, PhD, UC Davis, focused on critically important and understudied issues in RASopathies—feeding and gastrointestinal function. Family advocate Lindsay Homan, mother of a child with NS, Board member of the Noonan Syndrome Foundation, opened the session with her family's experience. She shared that while her son's diagnosis began with congenital heart disease, the complex and relentless GI complications, particularly dysmotility, are what defined much of his medical journey. Ms. Homan recounted the exhausting cycle of trial and error with feeding therapies and surgical interventions, amid ongoing setbacks as a major impact on quality of life. Her family story underscores the urgent need for research focused on GI dysmotility in children with a RASopathy.

Khalil El-Chammas, MD, MS, Cincinnati Children's Hospital, spoke on pediatric gastrointestinal neuromodulation. Acknowledging that the literature is sparse regarding gastrointestinal issues in RASopathies, he drew attention to reports highlighting the high prevalence of gastroesophageal reflux disease, vomiting, abdominal pain, and constipation. The suggestion that these findings are related to dysmotility and disorders of gut–brain interaction (DGBIs) is based on a limited number of reports, including a 1999 study by Shah et al. 1999. describing antroduodenal manometry indices in four patients with RASopathies compared with 10 controls. Treating dysmotility and DGBIs is based on a biopsychosocial model including neuromodulator medications, procedures (pyloric or anal Botox/dilation), and, in refractory cases, the consideration of gastrointestinal neurostimulation (gastric electrical stimulation, sacral nerve stimulation, percutaneous electrical nerve field stimulation). Further understanding of the enteric nervous system in dysmotility and DGBIs, specifically in RASopathies, will enable targeted approaches in diagnostics and therapies.

Abdul Elkadri, MD, Medical College of Wisconsin, discussed the use of intestinal organoid systems to study rare pediatric intestinal disorders. Intestinal organoid systems provide an opportunity for researchers to collect samples from patients with rare diagnoses, which can be converted into an epithelial stem cell model to recreate an exact genetic model without many of the issues encountered in mouse and cultured cell models. Though challenges exist, successes have been seen in disorders such as cystic fibrosis and TT7A-associated gastrointestinal defects and immunodeficiency syndrome. Gastrointestinal manifestations of RASopathies mainly manifest as motility issues, though autoimmune disorders have also been seen, including eosinophilic esophagitis and immune dysregulation such as inflammatory bowel disease and prolonged post-infectious diarrhea. Use of organoids provides an opportunity to recreate these clinical issues in a reproducible system that allows for drug testing and may provide a better understanding of the pathways and processes contributing to clinical complications.

John Fortunato, MD, MBA, Northwestern University Feinberg School of Medicine, spoke on the assessment and treatment of pyloric dysfunction in children with nausea and vomiting. RASopathies are associated with multiple gastrointestinal complaints, including feeding difficulties, oral aversion, vomiting, and constipation. As with all DGBI, the mechanisms underlying symptoms of nausea and vomiting are likely multifactorial. Diagnostic tools such as electrogastrography (EGG), pyloric impedance planimetry, and antroduodenal manometry can help differentiate a gastric myoelectrical cause from a smooth muscle etiology attributed to reduced pyloric distensibility or absence of effective antegrade peristalsis. These potential abnormalities underscore the notion that motility is defined both by the ability of the GI tract to effectively contract and, perhaps even more importantly, to relax, as indicated by the clinical efficacy of pyloric botulinum toxin and balloon dilatation (Osgood et al. 2023). Thus, the approach to nausea and vomiting in patients with a RASopathy should include a strategy to “hypothesize and synthesize,” in which symptom data and diagnostic tests are leveraged toward a systematic approach.

Miriam Bornhorst, MD, Lurie Children's Hospital of Chicago, discussed metabolic profiling in NF1. Patients with NF1 in general have decreased weight gain, decreased height, and evidence of increased metabolic rates, some of which is reversed when they are taking MEK inhibitor (MEKi) therapy to normalize RAS/MAPK signaling. Her research study reviewed weight-gain patterns in mice and patients receiving MEKi treatment, performing global metabolomics analysis of plasma samples using liquid chromatography-mass spectrometry. NF1 mice, as well as individuals with NF1, gained weight when treated with MEKi, and metabolomics data showed enrichment of pathways controlling long-chain fatty acid metabolism and phospholipid signaling/synthesis. Preliminary metabolomic analysis in her study provided data that MEKi leads to a decrease in lipid oxidation, which could account for increased weight gain during treatment.

Keir Shiels, MD, Great Ormond Street Hospital, London, discussed feeding avoidance in RASopathies. A patient questionnaire was distributed to patients and families in their multi-disciplinary RASopathy clinic to assess whether the clinic was meeting patients' clinical needs and offering the right support. Although feeding problems were ranked as a relatively high priority issue by families, only 45% had ever seen a dietician, and less than 10% had ever seen a psychologist with a feeding specialty. Nearly one third of families ranked feeding difficulties within their top three clinical priorities. While the literature may emphasize cardiac abnormalities of NS, everyday issues like feeding difficulties are overlooked, despite identical prevalence (50%–60%).

Dagmar Tiemens, PhD candidate, Radboud UMC, Nijmegen, spoke on Avoidant/Restrictive Food Intake Disorder (ARFID) and cognitive behavioral therapy (CBT) in RASopathies. Research has shown that feeding difficulties are currently one of the most significant challenges for children with NS and other RASopathies. They are at an increased risk of developing ARFID, which may arise from multiple contributing factors, including consequence aversion due to frequent vomiting, reduced appetite, diminished hunger cues, or decreased interest in food. A retrospective case–control study examined the effectiveness of CBT using the Dutch SLIK program (“swallow” in Dutch) in children with genetically confirmed NS and other RASopathies, compared to a matched group of children with ARFID but without a RASopathy. Both groups showed substantial improvement in intake, feeding skills, weight/height, and a reduction in tube dependence. No significant differences were found in outcomes or comorbid features. Therefore, CBT in conjunction with the SLIK program is equally effective in children with and without RASopathies.

## 4 ∣ Session 2: Systems—Neurodevelopment, Brain, and Behavior

Tamar Green, MD, Stanford University, moderated the session that focused on advances in neurodevelopmental and neurological studies of the RASopathies. Les Rogers, representative of CFC International, opened the session by sharing his daughter's journey with CFC syndrome, focusing on the impact of early-onset infantile spasms (West syndrome). Her clinical course was marked by hypsarrhythmia and a permanent loss of oral feeding during a period of uncontrolled seizures. While Vigabatrin eventually provided seizure control, MRI-confirmed secondary brain damage resulted in long-term motor impairments. Mr. Rogers highlighted that epilepsy affects 50%–60% of the CFCS population. He underscored the critical need for equitable access to specialized anti-epileptic therapies and further research into the genotype-phenotype correlations that drive severe neurological outcomes in the RASopathies.

Yong-Seok Lee, PhD, Seoul National University College of Medicine, reviewed recent studies investigating how mutations in the RAS/MAPK signaling pathway contribute to learning and memory impairments in RASopathy mouse models, with an emphasis on cell type-specific mechanisms. Dr. Lee described how *Nf1* haploinsufficiency primarily disrupts inhibitory synaptic transmission. In contrast, a *Ptpn11* mutation associated with NS (Shp2 D61G) affects excitatory synaptic function, revealing distinct RAS signaling networks across neuronal cell types (Shilyansky et al. 2010). He summarized how RNA-sequencing and molecular analyses showed neurofibromin enrichment in inhibitory neurons, and excitatory neuron-specific SHP2 coupling to RAS via Gab1/Grb2 (Ryu et al. 2019). Dr. Lee also highlighted recent findings in a CFCS model expressing a gain-of-function BRAF mutation (Braf K499E) in neural stem cells or astrocytes, but not neurons, which induced reactive astrocytes and severe memory deficits (Kang et al. 2025). At the mechanistic level, aberrant astrocytic calcium and ERK signaling mediated these impairments, which could be rescued by astrocyte-specific MEK inhibition or calcium suppression. Together, these findings demonstrate how distinct neuronal and glial RAS signaling mechanisms underlie cognitive dysfunction in RASopathies.

Rene Pierpont, PhD, University of Minnesota Medical Center, reported findings from a qualitative study exploring the perspectives of patients with RASopathies and their families regarding therapeutic approaches for neurodevelopmental and health care concerns. The study involved virtual focus groups with caregivers (*n* = 21) and young adults (n = 11) with a RASopathy. Pierpont's team applied a directed content analysis framework to analyze primary neurodevelopmental and mental health concerns, treatment history, and factors influencing service utilization. The analysis identified barriers to care including limited access to services, adverse medication effects, and insufficient provider experience or knowledge about RASopathies. Effectively managing neurodevelopmental and mental health symptoms often required family resilience and advocacy by patients and their caregivers. Additional emergent themes uncovered needs for provider training related to rare diseases, trauma-informed care, and improved community awareness regarding the RASopathies. Dr. Pierpont concluded by presenting a set of key actionable priorities for research, care delivery, and advocacy drawn from the perspectives and real-world experiences of both caregivers and patients with a RASopathy.

Julia Plank, PhD, Stanford University, a RASopathy Network Early Stage Investigator (ESI) awardee, presented her selected abstract examining myelin—the insulating material around nerve fibers—status in children with a RASopathy and discussed the challenge of identifying reliable in vivo markers for myelin. Dr. Plank's study examined whether a ratio of routinely acquired MRI scans (T1-weighted/T2-weighted, or T1w/T2w) could estimate cortical myelin content in children with a RASopathy compared to typically developing peers. She explained how T1w/T2w ratios were calculated using an automatic parcellation program (Freesurfer), controlling for age and sex as covariates. Her research found widespread reductions in T1w/T2w ratios across cortical regions in the RASopathies group, suggesting decreased myelin content. Dr. Plank noted that these findings align with preclinical evidence of disrupted oligodendrocyte development and emphasized the potential of T1w/T2w ratio mapping as a noninvasive biomarker for myelin integrity and therapeutic monitoring in the RASopathies.

Sara Pardej, PhD, Stanford University, introduced her ESI selected abstract evaluating academic achievement in youth with NS. Dr. Pardej's analyses revealed that youth with NS perform significantly lower (with large effects) on all assessed academic domains (including reading, math, and spelling) as compared to unaffected youth. The prevalence of Specific Learning Disorders among affected individuals ranged from 17% to 38%, considerably higher than the estimated 5% in the general population. Dr. Pardej also examined the distributions of IQ and academic achievement discrepancies across gene groups *(PTPN11*, *SOS1*, and Other variants) versus the unaffected group. While the *PTPN11* group distributions did not differ, significantly skewed IQ–Achievement distributions emerged in the *SOS1* and Other variants groups, implicating *SOS1* and Other variants as having larger impacts on academic ability than *PTPN11*. Finally, Dr. Pardej demonstrated that executive skills (processing speed, working memory, inhibitory control) are significant predictors of academic skills and account for ~1/3 to 1/2 of the variability in academic scores.

Nicole Weaver, MD, Cincinnati Children's Hospital Medical Center, addressed the prevalence and characteristics of sleep disorders in individuals with NS, CS, and CFCS. Analyzing data from three major centers, Dr. Weaver reviewed polysomnography (PSG) and sleep questionnaire results for 63 individuals with molecularly confirmed non-NF1 RASopathies. Among these, 27/63 (43%) individuals completed the PROMIS Sleep Disturbance survey. Overall, 37/63 (58%) had a diagnosis of obstructive sleep apnea and 16/63 (22%) had a diagnosis of periodic limb movement disorder. This included sleep disorders identified in 6 of 13 participants who had research-based PSG and no prior suspicion of a sleep disorder. Among the sub-cohort of pediatric patients with NS who completed the PROMIS surveys, reduced sleep efficiency and increased periodic limb movement index were associated with sleep disturbance. Dr. Weaver concluded that this work collectively suggests that medical sleep disorders are prevalent, potentially underdiagnosed, and associated with disturbed sleep in individuals with a RASopathy.

J. Elliott Robinson, MD, PhD, Cincinnati Children's Hospital Medical Center, presented findings from mouse model investigations showing that *Nf1* mutant mice exhibited heightened sensitivity to threatening visual stimuli, such as “looming discs” that mimic predator approach. These behavioral phenotypes were also observed in genetically engineered mice with a gain-of-function mutation in the *Mapk1* gene, confirming mediation by the RAS/MAPK signaling pathway. To identify relevant brain regions involved in threat hyperreactivity phenotypes, electrophysiology data showed that retinal ganglion cells in the eye are not more sensitive to light in the NF1 model mice, contrary to prior hypotheses. Dr. Robinson presented results from genetic experiments in which ERK was selectively activated in brain regions using a Cre-recombinase-inducible *Map2k1* gain-of-function mutant. While mice with ERK activation exclusively in the midbrain and cerebellum did not display behavioral differences, mice with ERK activation in the cerebral cortex and hippocampus had robustly increased sensitivity to looming visual threats. This effect was not caused by cortical interneurons given that activated MEK1 expression in forebrain GABAergic neurons did not alter threat reactivity. Dr. Robinson concluded noting that ongoing studies are investigating whether visual hypersensitivity involves cortical excitatory neurons or myelinating oligodendrocytes using *Nf1* conditional mutant mice. This research is directly relevant to understanding abnormal sensory processing and inhibitory control deficits affecting individuals with a RASopathy.

## 5 ∣ Session 3: Systems—Cardiovascular and Lymphatic Function

Bruce D. Gelb, MD, Icahn School of Medicine at Mount Sinai, moderated this session, focused on cardiovascular and lymphatic anomalies and therapies in the RASopathies. Tammy Bowers, president of the Noonan Syndrome Foundation, opened the session by sharing her son's journey, born with severe hypertrophic cardiomyopathy associated with NSML. Ms. Bowers described the critical nature of his early months, which involved a rapid progression to end-stage heart failure in the NICU. This clinical decline ultimately necessitated a life-saving heart transplant during his infancy. While the transplant has allowed her son to reach Age 15 to date and lead an active life, Ms. Bowers emphasized that transplantation is a “trade” rather than a cure, citing a medical history that includes numerous surgeries, chronic school absences, and ongoing risks of organ rejection and malignancy. Her narrative highlighted the high prevalence of cardiac abnormalities within the RASopathy population and underscores the need for research into medical therapies that could mitigate the need for such invasive surgical interventions and improve long-term quality of life.

Anton Bennett, PhD, Yale University, discussed the protein tyrosine phosphatase, SHP2, which is the product of the gene *PTPN11*, frequently altered in NS and NSML. Dr. Bennett stated that a key function of SHP2 is as a scaffold, which is enhanced due to both NS- and NSML-causal genetic variation. He discussed his group's work with the tyrosine kinase inhibitor, dasatinib, which rescues the hypertrophic cardiomyopathy (HCM) phenotype in an NSML mouse model at a low dose. Using “omics” approaches, the Bennett group showed that the protein PZR, which interacts directly with SHP2, is hyperphosphorylated in the NSML mouse heart, normalizes with dasatinib treatment, and that genetic elimination of PZR phosphorylation prevents the occurrence of HCM in the NSML mouse model. Dr. Bennett then showed data related to the identification of the homeobox factors IRX3 and IRX5 as key mediators, downstream from PZR, that inhibit signaling through the BMP10 growth factor (Perla et al. 2025).

Kathryn Chatfield, MD, PhD, Children's Hospital Colorado, discussed outcomes of treating cardiac and lymphatic complications in the RASopathies with pathway targeted therapies. First, Dr. Chatfield focused on the use of the MEKi trametinib in 48 patients, primarily to treat HCM and/or lymphatic issues, and also cardiac arrhythmias and seizures. She reviewed the use of trametinib across several RASopathy genes, relevant endpoints such as NT-proBNP as a marker of heart failure, and magnetic resonance lymphangiography, as well as side effects, primarily dermatological issues. Dr. Chatfield noted that she and others have assembled a detailed protocol for using trametinib for the RASopathies and are collecting retrospective data about treated patients. Lastly, Dr. Chatfield discussed the use of mTOR inhibitors (mTORi) for NSML-related HCM, for which retrospective data are also being collected.

Sarah Sheppard, MD, PhD, *Eunice Kennedy Shriver* National Institute of Child Health and Human Development (NICHD), spoke on precision medicine for RASopathy vascular anomalies. Her group has evaluated 356 patients with vascular and lymphatic anomalies, finding that approximately one third have germline or somatic alterations of the RAS/MAPK pathway (Li et al. 2023). Sheppard has modeled some of these in zebrafish, recapitulating lymphatic anomalies, and demonstrating that rescue with MEKi was superior to mTORi (Sheppard et al. 2023). Her group has gone on to develop additional zebrafish models with lymphatic phenotypes, which are being used as a drug screening platform, in the hope of finding more effective therapies for RASopathy-associated vascular and lymphatic anomalies.

Cristina Nuevo-Tapioles, PhD, NYU Grossman School of Medicine, presented her ESI-selected abstract on mitochondrial dysfunction in Costello syndrome. Dr. Nuevo-Tapioles discussed finding dysregulated mitochondrial function in striated muscle, both skeletal and cardiac, in a mouse model of CS with an *Hras* pathogenic allele. She found concomitant structural defects in CS muscle mitochondria. These mice were also less motorically active in adulthood. Dr. Nuevo-Tapioles then discussed a cell-based model using C2C12 skeletal muscle myoblasts to screen potential drug therapies to restore mitochondrial function in CS.

## 6 ∣ Session 4: RASopathies Genetics

This session highlighted different aspects of the RASopathies' genetic etiology and their potential phenotypic implications. Moderated by Karen W. Gripp, MD, Nemours Children's Health, the session started with a talk from Chuck Lawson, father of a child with Hiatt-Neu-Cooper neurodevelopmental syndrome (HINCONS), who shared the unique challenges of managing an ultra-rare RASopathy. He credits RASopathies Network for highlighting HINCONS and allowing him to connect with a growing number of families around the world that have received a similar diagnosis. HINCONS is caused by variants in the RAS-like *RALA* gene, presents with significant phenotypic variability including hypotonia, dyspraxia, and intellectual disability. Seizures are seen in about 60% of patients. Cardiac anomalies are rare in HINCONS. Mr. Lawson described his son's diagnostic journey, where Whole Exome Sequencing (WES) eventually identified the pathogenic variant after initial non-specific ASD and macrocephaly diagnoses. While the genetic diagnosis secured essential state-funded services, standard behavioral therapies have proven minimally effective for his son's profound communication deficits. Mr. Lawson's narrative underscores the urgent need for natural history studies and the development of tailored therapeutic strategies for emerging, ultra-rare RASopathy cohorts.

Anne Goriely, PhD, University of Oxford, UK, reported on the origin of de novo variants (DNMs) causing RASopathies and other dominant FGFR-related disorders. Unlike most spontaneous mutations, DNMs in the RAS/MAPK pathway show unusual features: they arise exclusively from the paternal germline (during spermatogenesis), occur at an increased frequency above the baseline mutation rate, and display a strong paternal age effect. These properties reflect a process termed “selfish selection,” whereby such variants confer a selective growth advantage to male germline stem cells, driving clonal expansion within the aging testis through mechanisms analogous to oncogenesis. Interestingly, because selfish selection occurs in all men as they age, accounting for the elevated birth prevalence of RAS/MAPK mutations, the contribution of classical gonadal mosaicism is low in RASopathies. As a result, the risk of recurrence to individual couples in subsequent pregnancies is much lower for RASopathies than for most other spontaneous genetic disorders. This insight should be incorporated into genetic counseling as it can provide reassurance to affected families when making future reproductive decisions (Wilkie and Goriely 2017).

Alessandro De Luca, PhD, IRCCS Casa Sollievo della Sofferenza, San Giovanni Rotondo, Italy, reviewed the prenatal features of RASopathies and the diagnostic yield of prenatal genetic testing. Increased nuchal translucency/nuchal fold (≥5mm), cystic hygroma, hydrops, ascites or pleural effusion, other lymphatic anomalies, and heart defects are suggestive of a RASopathy. Additional prenatal findings, such as polyhydramnios, renal anomalies, fetal macrosomia, mild ventriculomegaly, short/long bones, absent ductus venosus and increased head circumference are nonspecific, but supportive. Prenatal diagnostic testing is indicated with suggestive ultrasound findings and may be performed in parallel with karyotype analysis or copy number variation studies. Timely detection of a RASopathy prenatally allows for clinical decision-making. Clinical decisions may be informed not only by the particular causative gene, but also by the specific variant and its associated phenotype (Scott et al. 2021).

Emma Burkitt-Wright, MBChB, PhD, Manchester, UK, discussed the need to incorporate allele-specific information into clinical management. Focusing on NS, she reviewed the phenotypic differences associated with specific variants. While variant-specific information can be delineated in aggregate, it does not necessarily predict the course for an affected individual. Thus, screening and treatment recommendations for RASopathies are stratified by syndrome and disease-causing gene, if appropriate, rather than on a variant-specific level. Some exceptions apply; for example, embryonal rhabdomyosarcoma in CS has been reported recurrently only in association with variants of the HRAS p.Gly12 residue. Such observations may, however, be confounded by highly activating variants resulting in high early mortality not due to malignancy.

Kassidy E. Grover, University of Cincinnati College of Medicine, OH, presented her ESI-selected abstract on a mouse model for ERK2 activation. A knock-in mouse model *(Mapk1 A172V/*+) was created to study MAPK1-related RASopathy (MRR), a neurodevelopmental syndrome caused by gain-of-function variants in *MAPK1*. The mice exhibited gene dosage-dependent growth restriction, craniofacial abnormalities, increased oligodendrocyte precursor cell proliferation, myelin defects, and specific behavioral deficits, such as impaired spatial memory and heightened sensory reactivity. The observed neurological and molecular changes will be explored for therapeutic reversal using a brain penetrant MEKi.

## 7 ∣ Session 5: Predisposition to Malignancy

Marielle Yohe, MD, PhD, National Cancer Institute (NCI), moderated a session highlighting current understanding of the risk of malignancy in patients with RASopathies. The session began with a presentation by Lisa Schoyer, past president of RASopathies Network, who shared her perspective as the parent of a child with CS who developed rhabdomyosarcoma. Her son's early presentation included classic markers like the need for a g-tube and HCM (necessitating surgery); his oncology treatment was marked by severe complications, including a suspected cytokine storm and motor regression following standard chemotherapy. Ms. Schoyer's narrative highlighted the critical need for specialized oncology protocols that account for RASopathy-specific physiological sensitivities and comorbidities, such as GI dysmotility and respiratory issues. She concluded by advocating for the WHO International Classification of Functioning (ICF) framework, emphasizing a holistic, “whole-child” approach that prioritizes social participation and quality of life alongside intensive medical intervention.

The session continued with the work of Nancy Ratner, PhD, Cincinnati Children's Hospital, who has expertise in preclinical modeling of the RASopathy NF1. She discussed several of her lab's ongoing projects, including work on a genetically engineered mouse model of NF1-associated plexiform neurofibromas in which only Schwann cells lack neurofibromin. In this model, CD8+ T cell dependent inflammation was necessary for neurofibroma initiation and maintenance (Pundavela et al. 2024). Her lab also showed that the SHP2 inhibitor RMC-4550 decreased plexiform neurofibroma tumor volume in the NF1 mouse model (Ahmari et al. 2025).

Gina Ney, MD, PhD, NCI, described a study that used genomic ascertainment of electronic health record-linked exome data to quantify germline pathogenic/likely pathogenic variant prevalence, cancer prevalence, and survival in adults with non-NF1 RASopathies. This study showed a possible increase in cancer prevalence in *SPRED1* heterozygotes, with the most common cancer diagnosis being malignancy of the skin in this population. In addition, for some individuals with a RASopathy, cancer risk might not be elevated compared to the general population (Kim et al. 2024). Dr. Ney also described how cancer risk in the RASopathies is being evaluated prospectively through the NCI RASopathies Natural History study.

Marielle Yohe, MD, PhD, NCI, described functional characterization of germline *HRAS* variants with implications for genotype–phenotype correlation in CS. She outlined a battery of biochemical, cell biological, and model organism studies that her team is using to quantify both the impact of rare *HRAS* variants on signaling through the RAF/MEK/ERK pathway and tumorigenesis.

Maija Kiuru, MD, PhD, UC Davis, presented work on the risk of melanoma and melanocytic nevi (moles) in RASopathies. One of the strongest risk factors for melanoma is an increased number of nevi. Therefore, the risk of melanoma may be higher in RASopathies with an increased number of nevi, such as CFCS. Importantly, somatic mutations in the RAS pathway genes are key drivers of melanoma development, and two or more such mutations may act synergistically to activate the pathway.

## 8 ∣ Session 6: Therapeutic Pipeline

The final session of the symposium was moderated by Pau Castel, PhD, NYU School of Medicine. The session featured a keynote presentation by Frank McCormick, PhD, FRS, University of California San Francisco, discussing molecular analysis of the RAS/MAPK pathway. Dr. McCormick's lab had previously discovered several mechanisms that expand understanding of the MAPK pathway, both in cancer and the RASopathies, providing the rationale for development of novel therapies. He highlighted the importance of studying different members of the RAS GTPase family, given that many of these exhibit unique mechanisms of regulation and functions directly related to the pathophysiology of the RASopathies, such as MRAS, RRAS2, and RIT1. He closed by describing novel RAS inhibitors in the pipeline and how these could potentially be used for treatment of the RASopathies, including tumors associated with NF1.

Bruce D. Gelb, MD, Mount Sinai School of Medicine, provided an update on the clinical efforts to treat hypertrophic cardiomyopathy in the RASopathies. He presented the encouraging results of compassionate use treatment with the allosteric MEK1/2 inhibitor trametinib, on behalf of his colleagues (Wolf et al. 2025). Retrospective analysis showed that the use of trametinib in this patient population is associated with decreased mortality and morbidity with improved cardiac status. Reported side effects were manageable and not life-threatening. Based on these results, Dr. Gelb presented the design of an upcoming prospective study to assess the safety and efficacy of trametinib in children with a RASopathy and hypertrophic cardiomyopathy.

After this encouraging clinical data, a discussion panel of experts from academia and industry explored strategies to expedite the advancement and regulatory approval of RASopathy therapies, with particular attention to urgent indications such as HCM. The panel included Jaishri Blakeley, who brought expertise in the clinical and regulatory development of therapies for NF1, including the approvals of selumetinib and mirdametinib for plexiform neurofibroma; Saskia Brachmann (Novartis), with expertise in preclinical and translational research of agents targeting the RAS/MAPK pathway, including the MEKi trametinib; Matthew Traylor, PhD (Think Bioscience), with the perspective from early-stage biopharma exploring development of a small molecule therapeutic for Noonan syndrome; Bruce Gelb, MD (Mount Sinai), representing clinical investigators working to undertake prospective clinical trials to assess the efficacy of trametinib for RASopathy-associated HCM; and Kenneth Kern, MD, MPH, MS, MBEE, FACS (University of California, San Diego) on the design of clinical trials and regulatory considerations, particularly in the field of oncology. The panelists shared their views and recommendations on topics that included interactions with regulatory agencies, the need to develop prospective natural history studies, and the best approaches to design clinical trials that can result in novel therapies for the RASopathies, including the identification of well-defined endpoints, dose and schedules that can be tolerated, particularly for the pediatric population, and the need to establish rigorous biomarkers and target engagement. The panel also took several questions from the audience, including investigators and patient advocates.

In summary, the 9th International RASopathies Symposium brought together participants from 13 countries to learn and discuss the most current knowledge about the RASopathy syndromes. The conference facilitated sharing across medical and scientific disciplines, among advocates and expert practitioners in their conditions, and with representatives across the therapeutic landscape. RASopathy advocates are hopeful that sustained research funding and acceleration of drug repurposing efforts will translate to safe, approved treatments tailored to these rare conditions.

## Data Availability

Data sharing not applicable to this article as no datasets were generated or analysed during the current study.
